# Imaging features and prognostic value of ^18^F-FDG PET/CT detection of soft-tissue metastasis from lung cancer: a retrospective study

**DOI:** 10.1186/s12885-020-07080-0

**Published:** 2020-06-26

**Authors:** Tingting Xu, Xinyi Zhang, Shumao Zhang, Chunfeng Liu, Wenhui Fu, Chengrun Zeng, Yue Chen

**Affiliations:** 1 Department of Nuclear Medicine, the Affiliated Hospital of Southwest Medical University, Luzhou, Sichuan PR China 646000; 2 Nuclear Medicine and Molecular Imaging Key Laboratory of Sichuan Province, No 25 TaiPing St, Jiangyang District, Luzhou, Sichuan 646000 PR China; 3 Department of Respiratory Medicine, the Affiliated Hospital of Southwest Medical University, Luzhou, Sichuan PR China 646000

**Keywords:** ^18^F-FDG, Lung cancer, Soft-tissue metastasis, PET/CT

## Abstract

**Background:**

Soft-tissue metastasis (STM) is a relatively rare, but not exceptional, manifestation of lung cancer. The purpose of this study was to evaluate the imaging features of STM from lung cancer using fluorine-18 fluorodeoxyglucose positron emission tomography/computed tomography (^18^F-FDG PET/CT), and assess the impact of STM detected at baseline PET/CT on patient survival.

**Methods:**

Out of 4543 patients with lung cancer who underwent ^18^F-FDG PET/CT in our hospital between January 2013 and September 2018, 85 were diagnosed with STM (78 at baseline PET/CT and 7 at restaging PET/CT) and included in the imaging study. We conducted a comparative survival analysis between patients with stage 4 lung cancer with and without STM at baseline PET/CT (*n* = 78 in each group) and performed univariate and multivariate analyses to investigate the factors affecting the prognosis of lung cancer.

**Results:**

A total of 219 lesions were identified by ^18^F-FDG PET/CT: 215 were detected by PET and 139 by CT. Muscle STM were primarily found in the hip and upper limb muscle, whereas subcutaneous STM were mainly distributed in the chest, abdomen, and back. In 68 patients, STM were found incidentally during routine ^18^F-FDG PET/CT staging. Isolated STM were detected in 6 patients, whose tumor staging and treatment were affected by PET/CT findings. There were no significant differences in the 1-, 3-, and 5-year survival rates between patients with and without STM at baseline PET/CT. Brain and adrenal metastases, but not STM, were associated with poor prognosis of stage 4 lung cancer.

**Conclusions:**

We described the PET/CT imaging characteristics of STM from lung cancer, and confirmed that PET/CT can detect unsuspected STM to change the staging and treatment of some patients. Our analysis indicates that STM is not a useful prognostic indicator for patients with advanced lung cancer, while brain and adrenal metastases portend a poor prognosis.

## Background

Lung cancer is one of the most prevalent malignant tumors, and the leading cause of cancer-related death worldwide. In China alone, 700,000 new cases are diagnosed every year, resulting in 600,000 deaths per annum. Increasing environmental pollution has led to a surge in lung cancer incidence in recent years. Nearly 50% of patients are metastatic at diagnosis [1, 2]. Early diagnosis and treatment are essential for improving the survival of affected patients.

Soft-tissue metastasis (STM) refers to the growth of tumor cells in soft tissue that is not connected to the primary tumor or regional lymph nodes, and comprises metastases to skeletal muscle and subcutaneous tissue [3–5]. Although skeletal muscle and subcutaneous soft tissue account for more than 50% of the human body weight, STM is relatively rare [3–5]. Factors such as changes in local blood flow, presence of various proteases and inhibitors, high partial pressure of oxygen, changes in pH, pressure, and temperature, and local production of lactic acid are not conducive to the growth of tumor cells, making the soft tissue relatively resistant to the malignant penetration [4, 6–12]. Although infrequent, STM is still encountered in clinical practice and warrants greater attention of radiologists and clinicians [13].

Lung cancer is the most common primary tumor of STM, with adenocarcinoma being the most frequent histological variant [13–20]. The most common sites of distant metastasis of lung cancer include the bones, brain, adrenal glands, and liver, with the STM being much less common [3–5]. Usually, when lung cancer progresses to a certain extent, some of the tumor cells break away from the primary tumor and disseminate to remote sites through the bloodstream or lymphatic system [21–23]. If local tissue conditions are suitable, the cancer cells begin to divide and proliferate and gradually become metastatic foci [4]. A recent study showed that STM was associated with poor prognosis in lung cancer [7]. However, the prognostic value of specific organ metastases, including STM, is controversial and their effects on lung cancer have not been fully elucidated [24–28].

Magnetic resonance imaging (MRI) is the gold standard for imaging evaluation of soft-tissue diseases owing to its good soft tissue contrast [29]. However, it necessitates long acquisition times and is affected by movement artifacts [30]. Moreover, MRI is less sensitive than fluorine-18 fluorodeoxyglucose positron emission tomography/computed tomography (^18^F-FDG PET/CT) in identifying STM [31]. The latter technique uses a radioactive glucose analog, ^18^F-FDG, to image glucose uptake in tumors and adjacent healthy tissue, enabling improved localization and characterization of tumors. ^18^F-FDG PET/CT can reveal metabolic changes before the morphological abnormalities occur [15], and it has a high tumor-to-background FDG uptake ratio, allowing the detection of hidden STM [13, 32]. The widespread use of ^18^F-FDG PET/CT has led to increased detection of STM in various malignancies. However, reports on its use to identify STM from lung cancer are scarce, and most of them represent individual cases.

The purpose of this study was to explore the incidence and imaging characteristics of STM from lung cancer using ^18^F-FDG PET/CT. We also assessed the impact of ^18^F-FDG PET/CT findings on tumor staging and treatment, and evaluated the effect of STM detected at baseline PET/CT on the survival prognosis of lung cancer. Lastly, we studied the factors affecting the prognosis of lung cancer.

## Methods

### Patient selection

We retrospectively reviewed medical records of 4543 patients with lung cancer who underwent ^18^F-FDG PET/CT at the Affiliated Hospital of Southwest Medical University between January 2013 and September 2018. Based on the clinical, imaging, and histopathological data, 85 patients (1.87%) diagnosed with STM at baseline (78 patients) or re-staging (7 patients) ^18^F-FDG PET/CT were included in the imaging analysis. Sex, age, type, and maximum standardized uptake value (SUVmax) of primary tumor; clinical symptoms; location, size, shape, edge, density, number, SUVmax, and diagnostic method of STM; presence of concomitant distant metastases, including bone, liver, brain, adrenal gland, chest cavity (contralateral pulmonary metastases, pleural effusion/dissemination, and pericardial effusion/dissemination), and other rare metastases, were recorded for all study subjects. From the remaining 4458 subjects, we randomly selected 78 patients with TNM stage M1 lung cancer (regardless of T or N stage) without STM who underwent baseline PET/CT, to act as a control group for patients with STM at baseline PET/CT. The clinical features and distant metastasis of these patients were recorded.

In addition, we evaluated neurological symptoms and/or brain imaging data (MRI or contrast-enhanced CT) of all study subjects to assess brain metastasis. All patients were followed-up via our electronic medical system or telephone until September 2019, to determine health outcomes. Survival time was defined as the period from PET/CT imaging to death due to tumor-related disease.

### Inclusion and exclusion criteria

#### Patients with STM

The inclusion criteria were as follows: 1) underwent ^18^F-FDG PET/CT and diagnosed with STM for the first time; 2) primary lesion confirmed by puncture biopsy, fiberoptic bronchoscopy, or postoperative pathology. The exclusion criteria were as follows: 1) presence of lymphoma, malignant melanoma, neurofibroma, or other soft-tissue tumor; 2) soft-tissue lesions caused by direct infiltration from primary lesion or bone metastasis; 3) presence of lymph nodes, infection, inflammation, or post-biopsy reactions.

#### Patients without STM

The inclusion criteria were as follows: 1) underwent baseline ^18^F-FDG PET/CT and diagnosed with TNM stage M1 lung cancer (regardless of T or N stage) without STM; 2) primary lesion confirmed by puncture biopsy, fiberoptic bronchoscopy, or postoperative pathology. The exclusion criteria were as follows: 1) presence of other primary tumors; 2) lesions caused by direct infiltration from primary lesion.

### PET/CT scanning

^18^F-FDG was prepared using the Siemens Eclipse HD cyclotron and ^18^F-FDG automated chemical synthesis system, and had radiochemical purity of > 95%. The patients were asked to avoid strenuous physical activity the day before the scan, and fast for at least 6 h prior to intravenous administration of ^18^F-FDG (5.5 MBq/kg body weight) to ensure a blood glucose level of < 11.1 mmol/L. Following the injection, the patients rested for 40 min-1 h in the dark, drank 300–500 mL of lukewarm water, then underwent PET/CT scanning on a Philips Gemini TF 16 scanner after emptying the bladder. First, a 16-slice spiral CT scan was performed, ranging from the base of the skull to the middle upper thighs, with the arms raised above the head (120 kV, 100 mA, layer thickness 0.5 mm, matrix 512 × 512 pixels, window width 300–500 HU, window level 40–60 HU). If a patient was known to have abnormal lesions in the limbs, they were scanned from the top of the head to the feet, with the arms at the sides of the body. After CT was complete, three-dimensional PET was performed for 70–90 s per bed position, for a total of 7 bed positions. The resulting images were corrected by attenuation and reconstructed iteratively using the ordered subset expectation maximization method (3 iterations, 23 subsets, image size 144 × 144 (matrix)) to obtain transverse, coronal, and sagittal views of the PET/CT scans. Delayed imaging was performed 2 h after ^18^F-FDG injection, if necessary.

### Image analysis and diagnostic criteria

The images were analyzed for the presence of STM and other distant metastases by 3 experienced PET/CT physicians and a radiologist, using a combination of semi-quantitative analysis and visual assessment. Any disagreements were settled through negotiation. For the semi-quantitative analysis, a region of interest was drawn and the SUVmax was measured in the most intense area of focal ^18^F-FDG accumulation. The soft-tissue lesions were considered PET-positive if their ^18^F-FDG uptake was focal and greater than that of surrounding healthy muscle and subcutaneous soft tissue. CT-positive soft-tissue lesions were defined as obvious nodules, masses, or abnormal tissue structures. The location, density, maximum diameter, shape, edge, and SUVmax of each soft-tissue lesion were measured, and the number of STM metastases per patient was recorded. Other distant organ metastases were considered “positive” if their ^18^F-FDG uptake was greater than that of surrounding healthy tissue, or/and if abnormal density changes were noted. Combined with the literature [1, 3, 5, 13, 14, 17, 32], the final diagnostic criteria of STM and other distant organ metastases were histopathological or clinical evaluation (presence of symptoms or diffuse distribution of lesions), concordance between PET/CT results and those of other imaging methods (MRI or contrast-enhanced CT), and evidence of simultaneous remission/progression of primary and metastatic lesion on follow-up PET/CT or other imaging (MRI or contrast-enhanced CT). The patients were followed-up until September 2019.

### Survival analysis

To avoid possible bias due to previous treatment, only patients with baseline PET/CT scans were included in this analysis. Patients with unknown survival were excluded. Univariate and multivariate analyses were performed on the STM group and with STM as a variable (i.e. patients with and without STM combined). Lastly, the 1-, 3-, and 5-year survival rates were compared between patients with and without STM.

### Statistical analyses

All statistical analyses were performed in the statistical software R 3.6.0. Survival rates were estimated by the Kaplan-Meier estimator and compared between groups using the log-rank or Renyi-type test **(**the log-rank test was used when the proportional hazards assumption was satisfied; otherwise, a Renyi test was employed). Multivariate Cox proportional hazards regression models were applied to detect potential indicators of survival among patients with lung cancer. The significance level was set at *P* < 0.05.

## Results

### Clinical characteristics and PET/CT imaging features

Clinical characteristics of the 85 patients with STM of lung cancer are summarized in Table 1.

**Table 1:** Characteristics of the 85 patients with STM from lung cancer

Characteristic	Value	Result
Age (years)	Mean ± SD	61.8 ± 11.5
Sex	Male	58 (68.2%)
	Female	27 (31.8%)
Time PET/CT was performed	At baseline	78 (91.8%)
	During treatment	7 (8.2%)
Histology of lung cancer	ADC	51 (60%)
	SCLC	12 (14.1%)
	SqCC	11 (12.9%)
	NSCLC-NOS	6 (7.1%)
	ASCC	3 (3.5%)
	LCC	2 (2.4%)
First manifestation	STM	10 (11.8%)
	Primary tumor or other metastatic symptoms	75 (88.2%)
Manifestation of STM	Pain/swelling/nodule/mass	17 (20%)
	Asymptomatic	68 (80%)
Accompanied by other site metastasis?	No	6 (7.1%)
	Yes	79 (92.9%)
Location of STM	Skeletal muscle	41 (48.2%)
	Subcutaneous tissue	34 (40%)
	Skeletal muscle and subcutaneous tissue	10 (11.8%)
Diagnosis of STM	Histopathology	15 (17.6%)
	Clinical evaluation or imaging data	70 (82.4%)

*ADC* Adenocarcinoma, *ASCC* Adenosquamous carcinoma, *LCC* Large cell carcinoma, *NSCLC-NOS* Non-small cell lung carcinoma- not otherwise specified, *SCLC* Small cell lung cancer, *SD* Standard deviation, *STM* Soft-tissue metastasis, *SqCC* Squamous cell carcinoma

#### Number and imaging characteristics of STM

Muscle STM occurred in 41 cases and subcutaneous STM in 34 cases. In 10 of the patients, both types of STM were present. A total of 219 metastases were located by ^18^F-FDG PET/CT. Among them, 215 lesions were detected by PET (detection rate = 98.2%; median SUVmax = 6.12 (range 0.8–20.9)). CT identified 139 lesions (detection rate = 63.5%), out of which 109 were isodense and 30 were of low or slightly low density; 96 lesions were nodules or tissue masses, while 43 were accompanied by swelling and had unclear boundaries. Median lesion size was 2.12 cm (range 0.4–13.8).

There were 126 muscle metastases (57.5%), of which 125 were identified as hypermetabolic nodules by PET (detection rate = 99.2%; median SUVmax = 6.79 (range 2.1–20.9)) and 46 were identified as abnormal by CT (detection rate = 36.5%). There were 93 subcutaneous metastases (42.5%), of which 90 were identified as hypermetabolic nodules by PET (detection rate = 96.8%; median SUVmax = 5.36 (range 0.8–19.1)). All subcutaneous STM were identified as abnormal by CT (detection rate = 100%).

#### Location of STM

Muscle lesions were primarily distributed in the hip muscle, upper limb muscle, and dorsal muscle (Table 2), with the highest frequency in erector spinae, gluteus major muscle, and psoas muscle. Subcutaneous soft-tissue lesions were most commonly located in the chest and abdomen, followed by back, head and neck, hip, and, occasionally, in the extremities (Table 3).

**Table 2:** Distribution of skeletal muscle metastases

Location	No. of cases
Pelvic muscle	36 (28.6%)
Upper limb muscle	21 (16.7%)
Back muscle	20 (15.9%)
Abdominal muscle	16 (12.7%)
Pectoral muscle	14 (11.1%)
Head and neck muscle	11 (8.7%)
Lower limb muscle	8 (6.3%)
Total	126

**Table 3:** Distribution of subcutaneous tissue metastases

Location	No. of cases
Chest and abdomen	26 (28.0%)
Back	22 (23.7%)
Head and neck	20 (21.5%)
Pelvis	19 (20.4%)
Extremities	6 (6.4%)
Total	93

### Survival analysis of patients at baseline PET/CT

A total of 4 patients with STM and 5 patients without STM were lost to follow-up. Descriptive characteristics of the remaining patients are listed in Table 4.

**Table 4:** The demographic and clinical characteristics of patients with stage 4 lung cancer at baseline PET/CT

Variable	STM (***n*** = 74)	Non-STM (***n*** = 73)	Total (***n*** = 147)
	$\boldsymbol{N}\overline{/\boldsymbol{x}}\pm \boldsymbol{s}$	$\boldsymbol{N}\overline{/\boldsymbol{x}}\pm \boldsymbol{s}$	$\boldsymbol{N}\overline{/\boldsymbol{x}}\pm \boldsymbol{s}$
**Age (years)**	61.2 ± 11.8	62.8 ± 11.1	62.0 ± 11.5
**Sex**			
Female	24	25	49
Male	50	48	98
**Histology of lung cancer**			
ADC	44	44	88
SCLC	10	11	21
LCC	1	3	4
SqCC	11	12	23
ASCC	2	0	2
NSCLC-NOS	6	3	9
**SUVmax of lung cancer**	10.9 ± 5.7	12.2 ± 7.1	11.5 ± 6.4
**Bone metastasis**			
No	32	17	49
Yes	42	56	98
**Hepatic metastasis**			
No	59	54	113
Yes	15	19	34
**Brain metastasis**			
No	63	62	125
Yes	11	11	22
**Adrenal gland metastasis**			
No	58	51	109
Yes	16	22	38
**Metastasis within chest cavity**			
No	56	52	108
Yes	18	21	39
**Other distant metastasis**			
No	71	69	140
Yes	3	4	7
**SUVmax of STM**	5.8 ±4.0	/	/
**First manifestation**			
Primary tumor or other	65	/	/
metastasis			
STM	9	/	/
**Accompanied by other metastasis**			
Yes	69	/	/
No	5	/	/
**Survival situation**			
Death	65	67	132
Survival	9	6	15
**Median survival time (months)**	5.0 ± 12.7	6.0 ± 12.3	5.5 ± 12.4

*ADC* Adenocarcinoma, *ASCC* Adenosquamous carcinoma, *LCC* Large cell carcinoma, *NSCLC-NOS* Non-small cell lung carcinoma- not otherwise specified, *SCLC* Small cell lung cancer, *SqCC* Squamous cell carcinoma, *STM* Soft-tissue metastasis, *SUVmax* Maximum standardized uptake value

#### Univariate and multivariate analyses of overall survival rate in patients with STM as a variable (patients with and without STM combined)

Results of the univariate analyses demonstrated that adenocarcinoma (ADC) was associated with better prognosis, while small cell lung cancer (SCLC), SUVmax of lung cancer, and brain and adrenal gland metastases were all related with worse prognosis in patients with advanced lung cancer (Table 5). In contrast, presence of STM did not significantly affect the prognosis. Results of multivariate Cox proportional hazards model indicated that SCLC (HR = 2.178, 95% CI 1.044–4.541, *P* = 0.038), brain metastasis (HR = 2.470, 95% CI 1.240–4.921, *P* = 0.010), and adrenal gland metastasis (HR = 1.900, 95% CI 1.035–3.488, P = 0.038) were extremely effective at decreasing the lifespan of patients with advanced lung cancer (Table 6).

**Table 5:** Prognostic significance of potential indicators of overall survival in patients with lung cancer

Variable	***x***^**2**^/***Q***^**a**^	***P***-value	***P***-value (PH)^**b**^
Age (years)	0.302	0.583	0.823
Sex (male vs. female)	< 0.001	0.998	0.305
ADC	3.608	**< 0.001**	**0.004**
SCLC	4.916	**< 0.001**	**0.011**
SUVmax of lung cancer	4.885	**< 0.001**	**0.036**
STM	1.383	0.340	**0.032**
Bone metastasis	1.353	0.245	0.264
Hepatic metastasis	0.974	0.653	**0.042**
Brain metastasis	13.037	**< 0.001**	0.799
Adrenal gland metastasis	15.425	**< 0.001**	0.080
Metastasis within the chest cavity	0.096	0.756	0.873
Other distant metastasis	2.567	0.109	0.234

*ADC* Adenocarcinoma, *SCLC* Small cell lung cancer, *SUVmax* Maximum standardized uptake value, *STM* Soft-tissue metastasis

^a^Statistics for log-rank (satisfying the PH) or Renyi test (not satisfying the PH)

^b^Test for assumption of proportional hazard (PH)

Statistically significant *P*-values are highlighted in bold

**Table 6:** Multivariate Cox proportional hazards model for survival of patients with lung cancer

Variable^**a**^	B	SE	Wald	df	***P***-value	HR	95.0% CI for HR	
							Lower	Upper
**SCLC**	0.778	0.375	4.306	1	**0.038**	2.178	1.044	4.541
**Brain metastasis**	0.904	0.352	6.616	1	**0.010**	2.470	1.240	4.921
**Adrenal gland metastasis**	0.642	0.310	4.286	1	**0.038**	1.900	1.035	3.488

*CI* Confidence interval, *HR* Hazard ratio, *SCLC* Small cell lung cancer

^a^Variables selected by “forward (Wald)”

Statistically significant *P*-values are highlighted in bold

#### Univariate and multivariate analyses of overall survival rate in the STM group

Results of univariate analyses demonstrated that the number of STM did not affect the prognosis of patients with advanced lung cancer. ADC was associated with better prognosis, while SCLC, SUVmax of STM, and bone, brain, and adrenal gland metastases were all significantly related to worse prognosis in patients with STM from lung cancer (Table 7).

**Table 7:** Prognostic significance of potential indicators of overall survival in the STM group

Variable	***x***^**2**^/***Q***^**a**^	***P***-value	***P***-value (PH)^**b**^
**Age (years)**	0.211	0.646	0.281
**Sex (male vs. female)**	0.116	0.733	0.085
**First manifestation**	0.165	0.685	0.099
**Accompanied by other site metastasis**	2.159	0.142	0.531
**ADC**	2.588	**0.019**	**0.003**
**SCLC**	2.854	**0.009**	**0.043**
**SUVmax of lung cancer**	2.456	0.117	0.542
**SUVmax of STM**^**c**^	5.399	**0.020**	0.881
**Number of STM**	0.005	0.941	0.948
**Bone metastasis**	5.538	**0.019**	0.169
**Hepatic metastasis**	0.005	0.946	0.719
**Brain metastasis**	8.920	**0.003**	0.638
**Adrenal gland metastasis**	8.945	**0.003**	0.465
**Metastasis within the chest cavity**	0.153	0.696	0.963
**Other distant metastasis**	0.968	0.659	**0.036**

*ADC* Adenocarcinoma, *SCLC* Small cell lung cancer, *STM* Soft-tissue metastasis, *SUVmax* maximum standardized uptake value

^a^Statistics for Log-Rank (satisfying the PH) or Renyi test (not satisfying the PH)

^b^Test for assumption of proportional hazards (PH)

^c^Coding rules for SUVmax of STM: 1 = less than 5.8; 0 = great than or equal to 5.8

Statistically significant *P*-values are highlighted in bold

Furthermore, results of the multivariate Cox proportional hazards model indicated that SCLC (HR = 2.901, 95% CI 1.390–6.053, *P* = 0.005), bone metastasis (HR = 1.883, 95% CI 1.095–3.237, *P* = 0.022), and brain metastasis (HR = 2.638, 95% CI 1.316–5.288, *P* = 0.006) were extremely effective at decreasing the lifespan of patients with STM from lung cancer (Table 8). Patients with STM whose SUVmax was greater than or equal to 5.8 had 2.172 times the hazard faced by patients whose SUVmax of STM was less than 5.8 (95% CI 1.286–3.670, *P* = 0.004).

**Table 8:** Multivariate Cox proportional hazards model for survival of patients with STM from lung cancer

Variable^**a**^	B	SE	Wald	df	***P***-value	HR	95.0% CI for HR	
							Lower	Upper
**SCLC**	1.065	0.375	8.051	1	**0.005**	2.901	1.390	6.053
**SUVmax of STM**	0.776	0.268	8.404	1	**0.004**	2.172	1.286	3.670
**Bone metastasis**	0.633	0.276	5.239	1	**0.022**	1.883	1.095	3.237
**Brain metastasis**	0.970	0.355	7.481	1	**0.006**	2.638	1.316	5.288

*CI* Confidence interval, *HR* Hazard ratio, *SCLC* Small cell lung cancer, *STM* Soft-tissue metastasis; SUVmax, maximum standardized uptake value

^a^Variables selected by “forward (Wald)”

Statistically significant *P*-values are highlighted in bold

#### Overall 1-, 3-, and 5-year survival rates in the STM and non-STM group

**Fig. 1:**
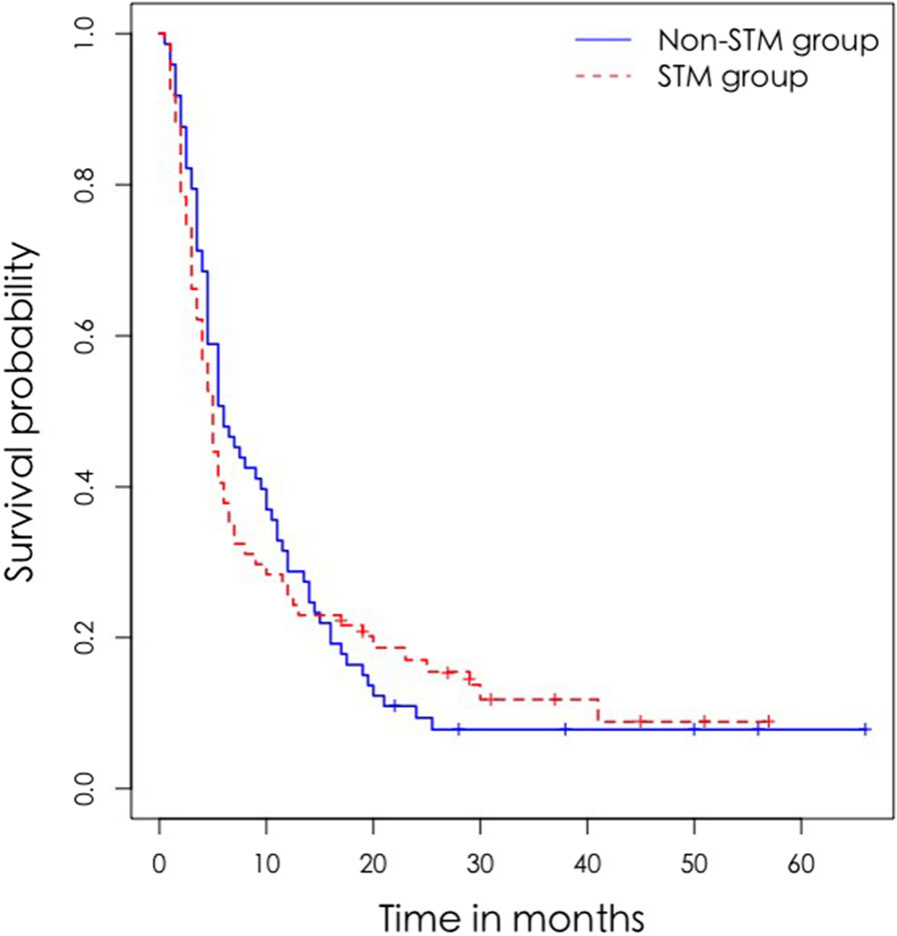
Survival of patients with lung cancer with or without STM

The Renyi test was not significant (Q = 1.372, *P* = 0.340), suggesting that STM was not related to prognosis in patients with advanced lung cancer (Table 9, Fig. 1).

**Table 9:** Comparison of overall survival rates between patients with and without STM from lung cancer

Follow-up time	STM (***n*** = 74)	Non-STM (***n*** = 73)	Q^**a**^	***P***-value
1 year	0.257 (0.174, 0.378)	0.288 (0.201, 0.413)		
3 years	0.171 (0.103, 0.284)	0.094 (0.046, 0.193)	1.372	0.340
5 years	0.118 (0.061, 0.230)	0.078 (0.035, 0.175)		

*CI* Confidence interval, *STM* Soft-tissue metastasis

^a^The Renyi test for comparison of survival of patients with or without STM from lung cancer

## Discussion

STM are defined as metastases to skeletal muscle and subcutaneous tissue [3–5]. Although soft tissue accounts for over 50% of the human body, and has abundant blood supply, it is a relatively rare site of metastasis. Factors such as changes to local blood flow; presence of various proteases and inhibitors; high partial pressure of oxygen; pH, pressure, and temperature changes; and local production of lactic acid are not conducive to the growth of tumor cells, making soft tissue relatively resistant to malignant penetration [4, 6–12]. Although infrequent, STM are still encountered in clinical practice and warrant greater attention of radiologists and clinicians [13].

Lung cancer is the most common primary malignant tumor leading to STM [13–17]. More than half of lung cancer cases are diagnosed at an advanced stage [1, 2]. The most common sites of distant metastasis include the bone, brain, adrenal glands, and liver, with STM being much less common [6, 29, 33]. Usually, when lung cancer progresses to a certain extent, some of the tumor cells break away from the primary tumor and disseminate to remote sites through the bloodstream or lymphatic system [21–23]. If local tissue conditions are suitable, the cancer cells begin to divide and proliferate and gradually become metastatic foci [4].

^18^F-FDG PET/CT can show metabolic changes before morphological abnormalities occur, and is used to screen for extra-pulmonary metastases in patients with lung cancer [15]. It is a whole-body imaging technique, with high tumor-to-background FDG uptake ratio, which allows detection of hidden STM [13, 32]. Despite these advantages, the use of ^18^F-FDG PET/CT to detect STM of lung cancer has not been widely researched. In previous studies, the prevalence of STM varied from 0.86 to 13% [13, 32]. In our review, we found that approximately 1.87% of patients with lung cancer had STM. Although this proportion is much lower than that for lung, liver, bone, or brain metastases, STM of lung cancer are not exceptional. Importantly, a more widespread use of ^18^F-FDG PET/CT may allow detection of previously undetected STM.

The median age and sex distribution in our study population was similar to that in previous studies [20, 21] of STM of lung cancer, indicating that the disease is the most prevalent in middle-aged and elderly males. Further, existing literature [16, 18–20] suggests that STM mostly occurs in patients with lung adenocarcinoma, which is consistent with our findings. Muscle metastasis is reportedly more common than subcutaneous metastasis, with a ratio of 1.2–3.3:1 [4, 5, 18]. This was also observed in the current study; the overall incidence of skeletal muscle STM was 60%, while that of subcutaneous STM was 51.8%, i.e. a ratio of 1.2:1.

SUVmax is the most widely used parameter to measure the uptake of a radiolabeled tracer by tumor tissue [34]. In this study, the median SUVmax of STM was 6.12 (range 0.8–20.9) while that of skeletal muscle and subcutaneous metastases was 6.79 (range 2.1–20.9) and 5.36 (range 0.8–19.1), respectively. The vast majority of metastatic lesions (98.2%) had high FDG metabolism, and could be detected by visual inspection of PET scans. A total of 80 muscle STM (36.5%) were missed by CT, which was probably related to poor density resolution of low-dose CT, and the isodensity of the lesions. The highest frequency of muscle metastases was in the hip, upper limb, and dorsal muscle, while subcutaneous metastases were mainly distributed in the chest, abdomen, and back. These findings are in line with those reported in the literature, and suggest that the staging of lung cancer should include a thorough examination of soft tissue [14, 16, 21, 35, 36].

**Fig. 2:**
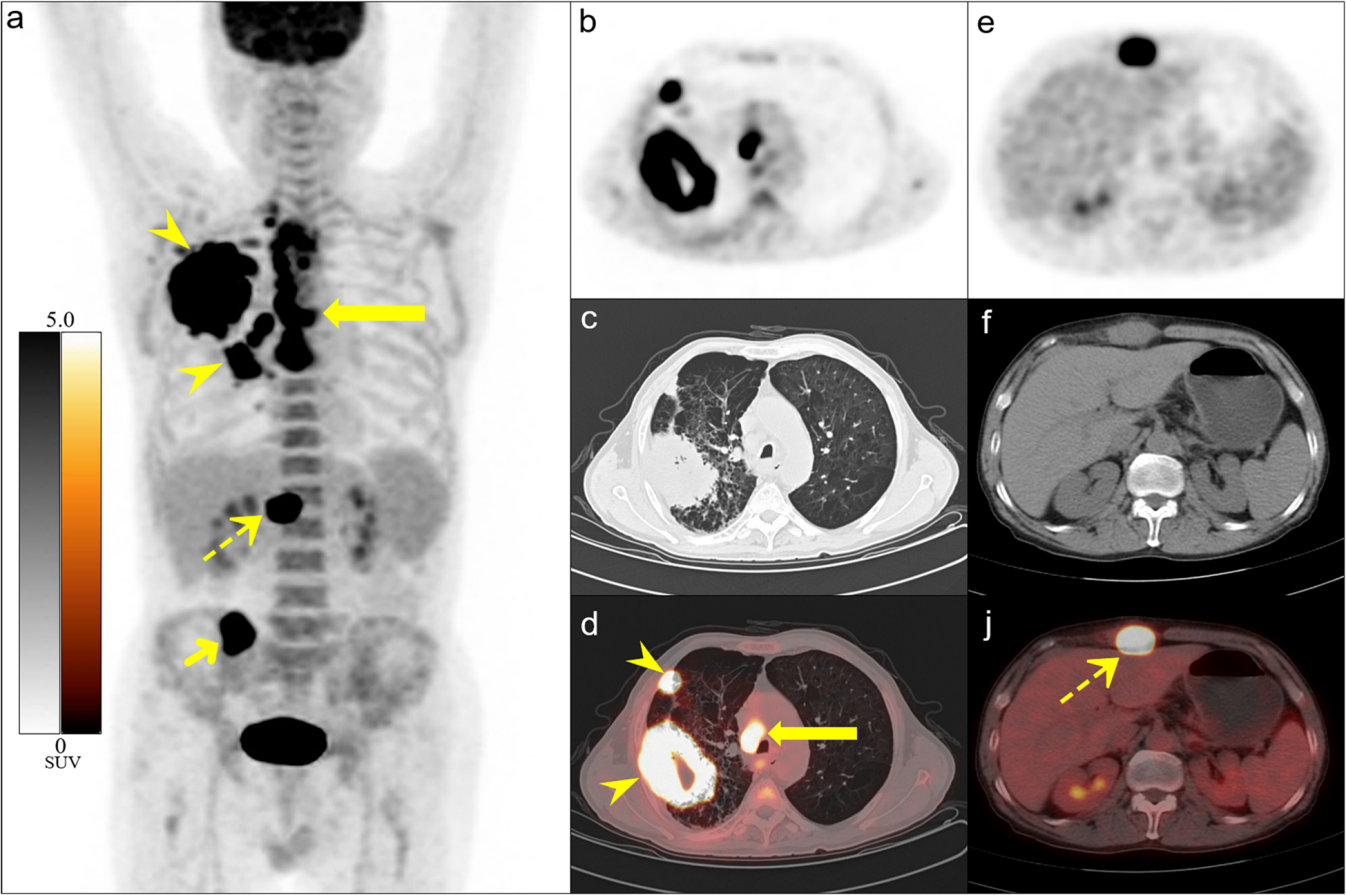
A case of lung adenocarcinoma with metastasis of right rectus abdominis as the first manifestation. A 64-year-old man presented with a 2-week history of a painful, tough mass in the upper abdomen, which was confirmed as metastatic adenocarcinoma by biopsy. ^18^F-FDG PET/CT imaging was performed to locate the primary tumor. Maximum intensity projection (MIP, **a**), chest axial images (**b**-**d**), and abdomen axial images (**e**-**g**) of PET/CT showed lesions in the upper lobe of the right lung (arrowheads), right rectus abdominis muscle (dotted arrows), multiple lymph nodes (long arrows) and right ilium (short arrow). Lung biopsy confirmed adenocarcinoma of the right lung. Therefore, a diagnosis of right lung cancer with lymph node, bone, and right rectus abdominis metastases was made. The patient survived for 6 months on palliative chemotherapy

Generally, STM are asymptomatic and easy to miss during clinical evaluation [13, 14]. Indeed, most of our patients (80%) did not present with symptoms related to their STM, and if ^18^F-FDG PET/CT had not been performed, the lesions would have likely remained undetected. If STM is the only metastasis, tumor staging and treatment might change dramatically. In 20% of the patients, the lesions were symptomatic, with local pain or swelling in muscle STM and painless masses in subcutaneous STM. Thus, in patients with lung cancer, unexplained muscle pain or subcutaneous nodules should raise suspicion of STM, and comprehensive physical and imaging examination should be conducted [29]. STM may also be the initial manifestation of lung cancer (Fig. 2), which was observed in 10 of our patients (11.8%). In such cases, in addition to active follow-up of medical history and physical examination, ^18^F-FDG PET/CT imaging should be performed as soon as possible to locate the primary tumor and ensure optimal patient management.

**Fig. 3:**
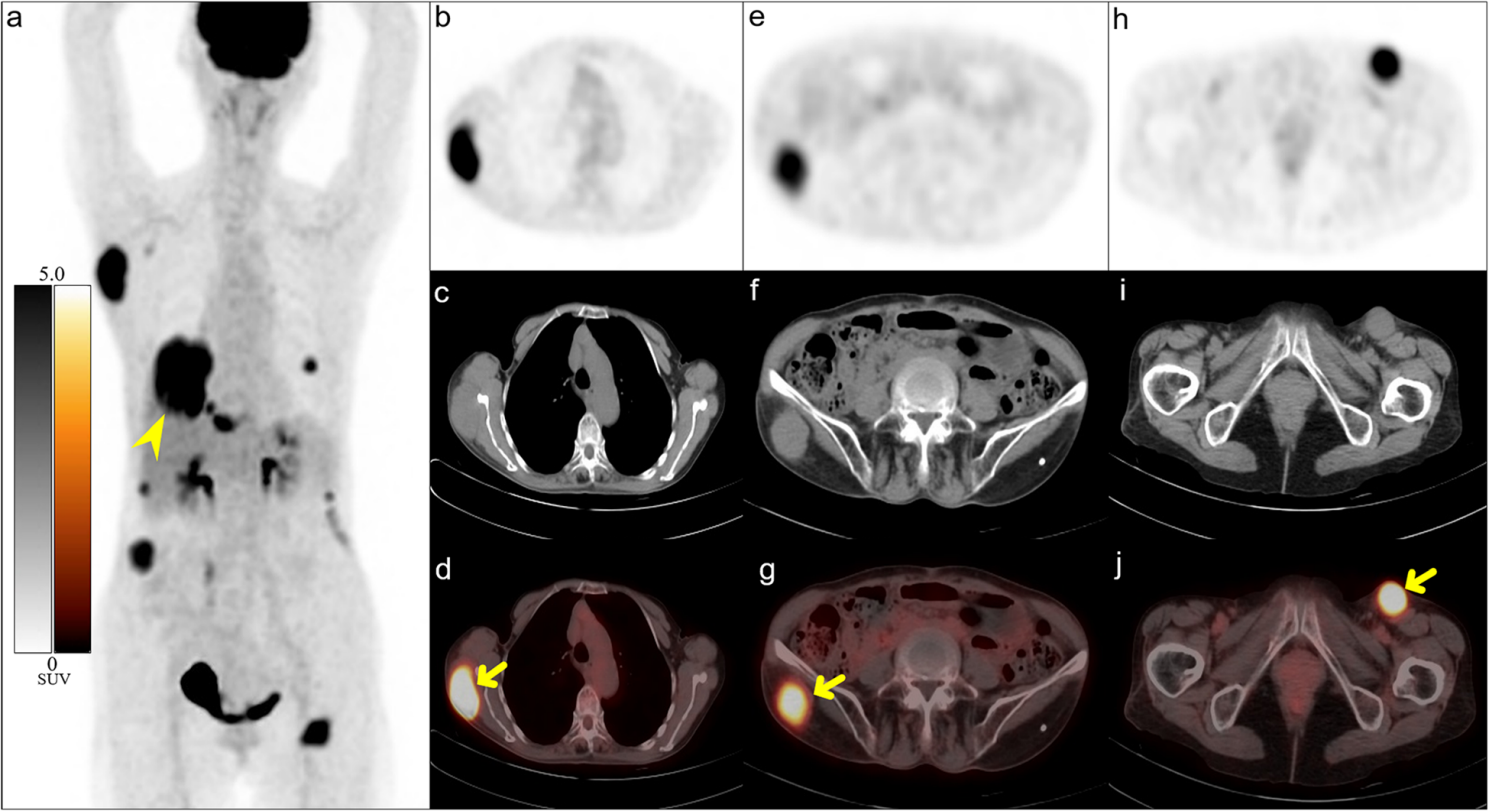
STM is the only manifestation of a small cell lung cancer. A 74-year-old woman presented with a 1-month history of a subcutaneous mass on the right side of her waist, which was confirmed as metastatic small cell carcinoma on biopsy. MIP (**a**) of ^18^F-FDG PET/CT showed a soft-tissue mass in the lower lobe of the right lung (arrowheads), with elevated FDG uptake (SUVmax = 8.4). MIP (**a**), chest axial images (**b**-**d**), and pelvis axial images (**e**-**j**) revealed multiple nodules and masses throughout subcutaneous tissue and skeletal muscle (short arrows) with increased FDG uptake (SUVmax = 7.5). Subsequently, lung biopsy confirmed small cell lung cancer of the right lung. After 11 months of palliative chemotherapy, the patient died of respiratory failure

**Fig. 4:**
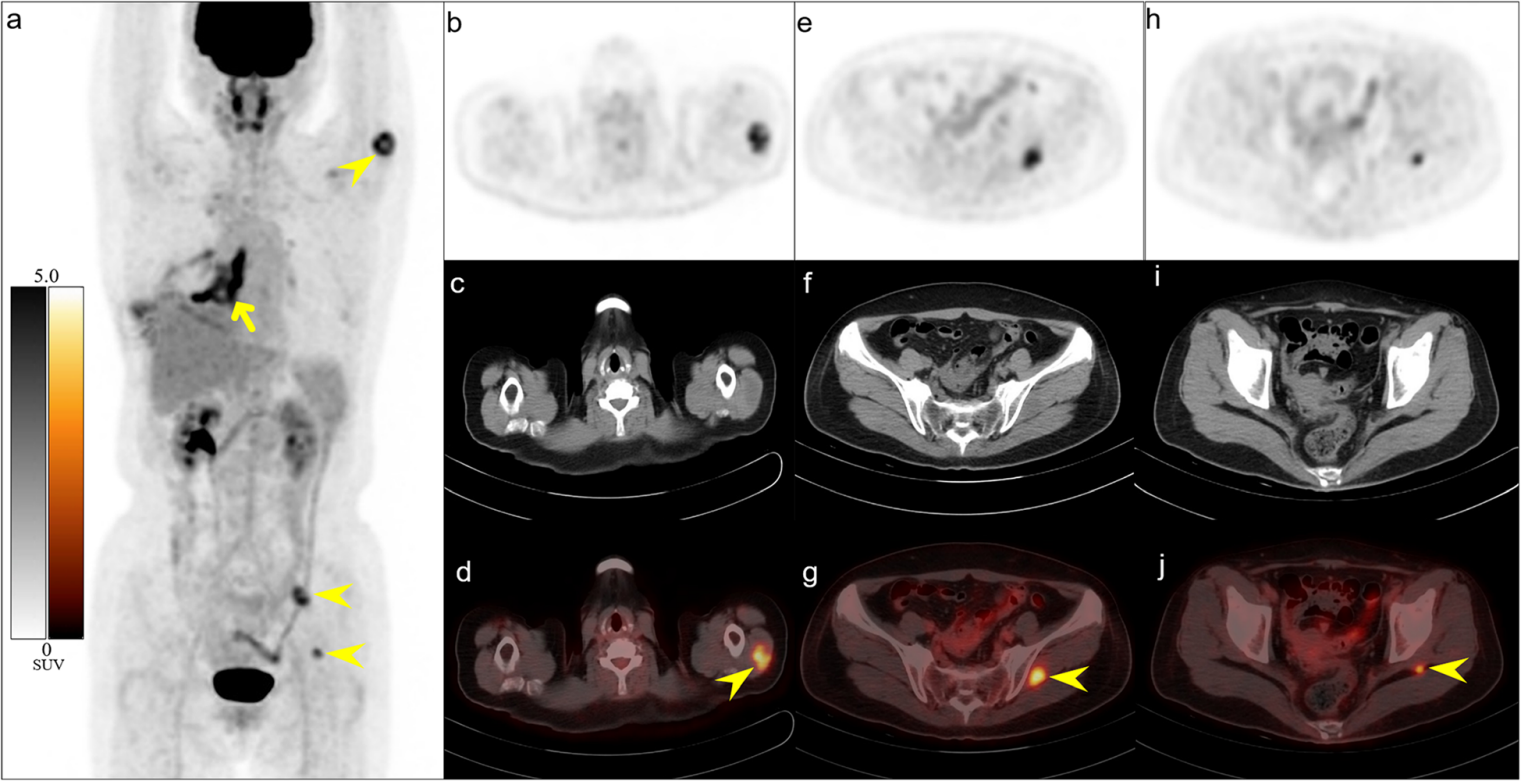
STM changed the postoperative stage of a lung squamous cell carcinoma. A 62-year-old woman was referred to our hospital with a 2-month history of cough. Squamous cell carcinoma of the lower lobe of the right lung was diagnosed by chest CT and lung biopsy. The general condition of the patient was good, and no metastases were found in head MRI or thoracic and abdominal CT. The patient underwent surgical resection and received adjuvant chemotherapy after the operation. Three months later, the patient underwent ^18^F-FDG PET/CT to assess treatment efficacy. MIP (**a**) showed increased ^18^F-FDG uptake (SUVmax = 3.8) in the operative area of the right lung (short arrows). MIP (**a**), axial images of neck and pelvis (**e**-**j**) revealed localized reduced-density nodules in the left deltoid muscle, left gluteus medius muscle, and left gluteal muscle (arrowheads), with FDG uptake (SUVmax = 8.0). Therefore, a diagnosis of multiple STM after lung cancer resection was considered. The patient was treated with palliative radiotherapy and chemotherapy to control the disease

Most patients with STM of lung cancer display multiple organ and lymph node metastases, and since metastasis mostly occurs in patients with a high degree of malignancy, their prognosis is poor [4, 5, 16, 33]. Among the 85 patients in our study, 79 had extensive metastatic diseases. ^18^F-FDG PET/CT detection of additional STM does not have a significant effect on the staging of lung cancer patients with extensive metastases, but it can help delineate the target area for local radiotherapy [19]. ^18^F-FDG PET/CT could also guide biopsies of soft-tissue lesions, which usually occur in superficial areas. A small proportion of patients (7.1%) showed solitary STM on ^18^F-FDG PET/CT (Figs. 3 and 4), which was the only manifestation of metastatic disease. ^18^F-FDG PET/CT results completely changed tumor staging, treatment plan, and prognosis of these patients.

Understanding the impact of specific organ metastases, including STM, on the survival of patients with advanced lung cancer is crucial for appropriate treatment and follow-up strategies. However, the effect of different metastatic organs on the prognosis of lung cancer has not been fully elucidated and the prognostic value of STM in advanced lung cancer remains controversial. A recent study by Kanaji et al. [7] showed that STM was associated with poor prognosis and worse response to treatment in lung cancer. Fei-Yu Niu et al. [1] demonstrated that survival time of patients with uncommon metastases from lung cancer (including STM) was significantly shorter than that of patients with common metastases. In other studies, STM did not impact the prognosis [24]. Herein, although the median survival of patients with STM (5 months) was shorter than that of those without STM (6 months), the 1-, 3-, and 5-year survival rates did not differ significantly between the groups (*P* = 0.340), suggesting that STM does not affect the prognosis of patients with advanced lung cancer. Nevertheless, detection of STM by ^18^F-FDG PET/CT can be used as an indicator of disease status, because it provides accurate information about tumor load, which could impact treatment decisions. In addition, multivariate analysis showed that SUVmax of STM was associated with poor survival in the STM group, suggesting that SUVmax of STM reflects disease malignancy. When presence of STM was used as a variable, brain and adrenal metastases were related with poor survival. Previous studies investigating whether specific metastatic organs (other than STM) affect survival of patients with lung cancer yielded contrasting conclusions. In Sorensen et al. [37] brain metastasis was an independent prognostic factor in patients with lung cancer, which is consistent with our results, and may be explained by irreversible nerve injury caused by brain metastasis [38, 39]. In other studies [24, 40, 41], bone metastasis portended poor prognosis, possibly owing to bone-related events such as pathological fractures, spinal cord compression, and malignant hypercalcemia [42]. Liver metastasis is also associated with shorter survival in patients with lung cancer [24, 40, 43–48]. Since the liver is an important part of the immune system, metastatic cancer cells may inhibit the immune response and induce immune tolerance [49, 50]. In Tamura et al. [2] and Abbas et al. [24], adrenal metastasis implied poor prognosis, which is consistent with our findings. However, adrenal metastases rarely show severe symptoms and their exact cause is unclear [51]. Some researchers believe that specific organ metastases do not affect the prognosis of lung cancer [25–28]. And some researchers [28, 52] propose that the increase in the number of metastatic organs reflects the ability of tumor cells to adapt to varying tissue microenvironments, resulting in the emergence of drug resistance and shortening of survival time. In our retrospective analysis, we did not assess the impact of the number of metastatic organs on advanced lung cancer. Larger scale studies are needed to confirm the effects of specific organ metastases, and the number of metastatic organs, on patients with this disease.

### Limitations

First of all, our study was retrospective and spanned a relatively long period of time. Diagnosis of metastatic organs mostly depends on clinical evaluation and imaging data, and most STM and other distant metastases lacked detailed pathology. In fact, only 17.6% of patients were confirmed to have STM by histopathology. While in line with patient care standards (most metastases do not need pathological diagnosis), it might have caused deviation in the results [13, 14, 17]. In addition, a variety of physiological and pathological factors, including hyperactivity, infectious/inflammatory processes, post-surgical reactions, primary soft-tissue tumors, and lymphoma, may increase ^18^F-FDG uptake in soft tissue [18, 53], leading to false positive results. Conversely, factors that decrease ^18^F-FDG uptake by soft tissue (small lesions, tumors with low metabolic activity, elevated blood glucose levels, etc.) could lead to false negative results.

Second, the density resolution of low-dose CT for attenuation correction is relatively poor, which may have failed to detect lesions with small density changes.

Third, the vast majority of our patients were scanned from the base of the skull to the middle upper thighs, which is not a true whole-body (TWB) scan. In previous studies, ^18^F-FDG PET/CT detected limb STM in 51.8% (9/12) - 75% (14/27) patients with STM of lung cancer [3, 54], and approximately 11.7–46.8% of STM lesions located in the extremities [3, 5, 13, 32]. Nguyen et al. [3] used TWB PET/CT to evaluate STM and found that approximately 46% of the lesions occurred outside the field of vision of limited whole-body (LWB) PET/CT. In our study, 14.1% (12/85) patients with limb STM, 15.9% (35/219) of STM were located in the extremities. These proportions are lower than those reported in the literature, suggesting that many lesions outside the LWB scan range may have been missed. Missed diagnosis of limb metastases can underestimate the extent of STM, leading to under-staging and mis-management of the disease. Newer PET/CT technology allows fast whole-body scanning without affecting imaging accuracy. In our future work, we will gradually adopt the whole-body approach to PET/CT imaging (from the top of the head to the soles of the feet) to prevent missed lesions.

Fourth, some preclinical brain metastases might have been missed as not all patients with lung cancer underwent head MRI or contrast-enhanced CT, possibly affecting the results of the study. In addition, not all patients underwent thoracic and abdominal CT enhancement. Therefore, we could not compare the diagnostic performance of PET/CT and contrast-enhanced CT in the detection of STM.

Finally, due to the small number of SCLC cases, we were unable to reliably compare patients with SCLC and NSCLC. Therefore, we did not study the two groups separately. Further, since not all patients received systematic treatment, and, in many cases, the information about treatment was limited, we did not analyze the effects of various treatments in this study.

## Conclusions

STM is a relatively rare, but not exceptional, manifestation of lung cancer. There are few studies on ^18^F-FDG PET/CT detection of STM from lung cancer, and most of the existing data is derived from case reports. Thus, our results make a valuable contribution to the literature. We assessed the incidence and imaging characteristics of STM from lung cancer using ^18^F-FDG PET/CT, which will help clinical and nuclear medicine doctors deepen their understanding of the disease and guide timely assessment of patients with lung cancer. Further, we confirmed that ^18^F-FDG PET/CT can detect unsuspected STM, and thus change the staging and treatment in some cases. Although PET/CT-detected STM were not a useful prognostic indicator, other metastatic diseases, such as brain and adrenal gland metastases, were associated with poor prognosis of advanced lung cancer.

## Data Availability

The datasets and materials during the present study are available from the corresponding author on reasonable request.

## Abbreviations

^18^F-FDG: Fluorine-18 fluorodeoxyglucose
PET/CT: Positron emission tomography/computed tomography
STM: Soft-tissue metastasis
SUVmax: Maximum standardized uptake value
MRI: Magnetic resonance imaging
TNM: Tumor-node-metastasis
SD: Standard deviation
ADC: Adenocarcinoma
SCLC: Small cell lung cancer
SqCC: Squamous cell carcinoma
NSCLC: Non-small cell lung carcinoma
NSCLC-NOS: Non-small cell lung carcinoma- not otherwise specified
ASCC: Adenosquamous carcinoma
LCC: Large cell carcinoma
HR: Hazard ratio
CI: Confidence interval
PH: Proportional hazards
MIP: Maximum intensity projection
TWB: True whole-body
LWB: Limited whole-body
